# Targeting GD2-Positive Tumor Cells by Pegylated scFv Fragment–Drug Conjugates Carrying Maytansinoids DM1 and DM4

**DOI:** 10.3390/cimb45100512

**Published:** 2023-10-05

**Authors:** Daniel V. Kalinovsky, Irina V. Kholodenko, Elena V. Svirshchevskaya, Alexey V. Kibardin, Dmitry Yu. Ryazantsev, Fedor N. Rozov, Sergey S. Larin, Sergey M. Deyev, Roman V. Kholodenko

**Affiliations:** 1 Department of Immunology, Shemyakin-Ovchinnikov Institute of Bioorganic Chemistry, Russian Academy of Sciences, 16/10, Miklukho-Maklaya St., Moscow 117997, Russia; dcalinovschi@yahoo.com (D.V.K.); esvir@mail.ibch.ru (E.V.S.); d.yu.ryazantsev@gmail.com (D.Y.R.); frozov@yandex.ru (F.N.R.); biomem@mail.ru (S.M.D.); 2 Laboratory of Cell Biology, Orekhovich Institute of Biomedical Chemistry, 10, Pogodinskaya St., Moscow 119121, Russia; 3 Laboratory of Molecular Immunology, D. Rogachev Federal Research Center of Pediatric Hematology, Oncology and Immunology, 1, Samory Mashela St., Moscow 117997, Russia; alexey.kibardin@gmail.com (A.V.K.); sergei_larin@mail.ru (S.S.L.); 4 Laboratory of Molecular Pharmacology, Institute of Molecular Theranostics, Sechenov First Moscow State Medical University, 8-2, Trubetskaya St., Moscow 119992, Russia; 5 “Biomarker” Research Laboratory, Institute of Fundamental Medicine and Biology, Kazan Federal University, 18 Kremlyovskaya St., Kazan 420008, Russia; 6 Real Target LLC, Miklukho-Maklaya St., 16/10, Moscow 117997, Russia

**Keywords:** antibody fragments, ganglioside GD2, pegylation, multimerization, maytansinoids, immunotherapy, cancer, drug conjugates, ADC, FDC, GD2-positive tumors

## Abstract

Oligomerization of antibody fragments via modification with polyethylene glycol (pegylation) may alter their function and properties, leading to a multivalent interaction of the resulting constructs with the target antigen. In a recent study, we generated pegylated monomers and multimers of scFv fragments of GD2-specific antibodies using maleimide–thiol chemistry. Multimerization enhanced the antigen-binding properties and demonstrated a more efficient tumor uptake in a syngeneic GD2-positive mouse cancer model compared to monomeric antibody fragments, thereby providing a rationale for improving the therapeutic characteristics of GD2-specific antibody fragments. In this work, we obtained pegylated conjugates of scFv fragments of GD2-specific antibodies with maytansinoids DM1 or DM4 using tetravalent PEG-maleimide (PEG4). The protein products from the two-stage thiol–maleimide reaction resolved by gel electrophoresis indicated that pegylated scFv fragments constituted the predominant part of the protein bands, and most of the scFv formed pegylated monomers and dimers. The conjugates retained the ability to bind ganglioside GD2 comparable to that of the parental scFv fragment and to specifically interact with GD2-positive cells. Both induced significant inhibitory effects in the GD2-positive B78-D14 cell line, in contrast to the GD2-negative B16 cell line. The decrease in the B78-D14 cell viability when treated with scFv-PEG4-DM4 was more prominent than that for scFv-PEG4-DM1, and was characterized by a twofold lower half-maximal inhibitory concentration (IC50). Unlike the parental scFv fragment, the product of scFv and PEG4 conjugation (scFv–PEG4), consisting predominantly of pegylated scFv multimers and monomers, induced direct cell death in the GD2-positive B78-D14 cells. However, the potency of scFv–PEG4 was low in the selected concentration range, thus demonstrating that the cytotoxic effect of DM1 and DM4 within the antibody fragment–drug conjugates was primary. The suggested approach may contribute to development of novel configurations of antibody fragment–drug conjugates for cancer treatment.

## 1. Introduction

Ganglioside GD2 is a prominent tumor-associated carbohydrate antigen (TACA) expressed by several types of tumors, including neuroblastoma, glioma, breast cancer, sarcomas, small cell lung cancer, and melanoma [1]. High and homogeneous GD2 expression on many tumors has made it a perspective TACA in various immune therapies aimed to promote both an active and passive anti-tumoral immune response [2]. The GD2-specific monoclonal antibodies dinutuximab and naxitamab are the only TACA-directed therapeutics that have been granted regulatory approval. Despite clinical efficiency of these drugs, notable limitations exist regarding their application.

One of the main limitations is the poor penetration of these full-length antibodies into GD2-positive solid human tumors [2,3]. Two strategies could be most promising in increasing tumor penetration. The first is to enhance the cytotoxic activity of the therapeutic molecules that reach the tumor site. In an earlier work, we developed GD2-targeted antibody–drug conjugates (ADCs) which significantly amplified the therapeutic effect of the parent full-length antibodies [4]. The conjugates of chimeric GD2-specific antibodies ch14.18 and the microtubule-depolymerizing drugs monomethyl auristatin E (MMAE) or monomethyl auristatin F (MMAF) showed a high and selective cytotoxicity in a broad panel of GD2-expressing tumor cell lines, and also strongly inhibited the growth of solid tumors in mouse models of melanoma and lymphoma [4]. The second strategy for overcoming the poor tumor penetration of full-length antibodies is to replace them with smaller antigen-binding molecules. Full-length antibodies must overcome a number of biological barriers in order to reach tumor sites, such as an insufficient blood supply to the tumor, the vascular endothelium, tumor interstitial pressure, and finally the tumor stroma. This typically results in less than 1% of the administered ADC dose per gram of tissue accumulating in human solid tumors [5]. Due to their smaller size, antibody fragments pass through the walls of blood vessels and diffuse into the tumor faster, and are more evenly distributed throughout it [6]. All major antibody fragment formats can be employed and are being studied in this aspect, namely minibodies, Fab fragments, diabodies, scFv fragments, and nanobodies, as well as lower-molecular-weight antigen-binding peptides [3,7,8]. From this point of view, the development of antibody fragment–drug conjugates (FDC) seems reasonable as an approach combining both aforementioned strategies to improve the poor tumor penetration of GD2-targeted drugs.

In our recent work, GD2-directed FDCs were generated based on minibodies and scFv fragments, and both of these carried variable antibody domains identical to those of dinutuximab. The minibodies and scFv fragments were conjugated to MMAE or MMAF and showed a good stability, high binding to GD2 antigen, and selective cytotoxic effects in GD2-positive but not GD2-negative cell lines [9]. However, both FDCs and their parental antibody fragments share a key limitation for therapeutic use of their own, that being their short blood half-life. In humans, full-length IgG antibodies manifest a blood half-life from 7 to 21 days, while antibody fragments are eliminated within minutes to hours [10].

Pegylation is one of the strategies for improving the pharmacokinetic and pharmacodynamic properties of antibody fragments [11,12]. Protein modification by PEG increases their hydrodynamic size, reduces the hepatic clearance and absorption by the reticuloendothelial system, and significantly increases the enhanced permeation and retention (EPR) effect. Pegylated antigen-binding molecules are capable of accumulating to a greater extent within the foci of tumor growth compared to unmodified proteins interacting predominantly with the antigenic markers on tumor cells [13,14,15]. Pegylation may be used to oligomerize antibody fragments [16], which, as a rule, leads to a multivalent interaction of the resulting constructs with the target antigen.

In an earlier study, we conjugated GD2-specific scFv fragments with the engineered C-terminal cysteine to divalent or tetravalent maleimide-activated PEG molecules, yielding predominantly pegylated scFv multimers and monomers. We evaluated the enhanced antigen-binding properties and demonstrated a more efficient tumor uptake of the reaction products in a syngeneic GD2-positive mouse cancer model, compared to the parent scFv fragment, thus providing the rationale for improving the therapeutic characteristics of GD2-specific antibody fragments via multimerization [17]. In this study, the same conjugation approach was used for the generation of a novel FDC, in which the tetravalent PEG-maleimide backbone links the GD2-specific scFv fragment with the thiol-containing cytotoxic drugs maytansinoids DM1 or DM4. The stability, antigen-binding properties, and cytotoxic effects of these FDCs were analyzed in GD2-positive and -negative tumor cell lines.

## 2. Materials and Methods

### 2.1. Generation of Pegylated Antibody Fragment–Drug Conjugates

**Figure 1:**
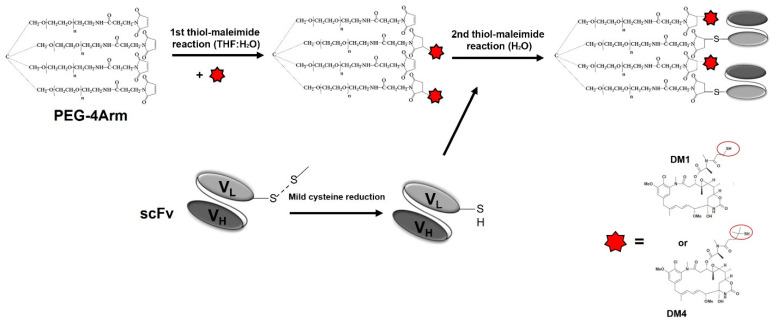
Reaction scheme for generating pegylated antibody fragment–drug conjugates.

The scFv fragment of the GD2-specific antibody 14.18 was produced and purified as described previously [17]. For the generation of the pegylated conjugates of scFv fragments with thiol-containing maytansinoids DM1 and DM4 (MedChemExpress LLC, Monmouth Junction, NJ, USA), a tetrafunctional 4-arm PEG maleimide with a 10 kDa molecular weight (4arm PEG Maleimide, Plano, TX, JenKem Technology, Plano, TX, USA) was used, which, for simplicity, is further addressed as PEG4. The conjugation was performed using two sequential thiol–maleimide reactions (Figure 1). For the binding of the DM1 or DM4 thiol groups with the PEG4 maleimide groups, the first thiol–maleimide reaction was carried out in an aqueous-organic solution consisting of 25% buffer A (20 mM phosphate buffer supplemented with 50 mM of NaCl and 10 mM of ethylenediaminetetraacetic acid (EDTA) at pH 6.0) and 75% tetrahydrofuran (THF). The maytansinoid: PEG4 molar ratio was 12:1. The reaction mixture consisting of DM1 (or DM4) and PEG4 was incubated for 2 h at 37 °C and with agitation; after this, it was lyophilized and reconstituted in distilled water, followed by filtration through Amicon Ultra-4 centrifugal 3 kDa filters and through 0.22 μm membrane filters (both from Merck, Rahway, NJ, USA) to remove the free drugs which were not conjugated to PEG4.

A mild reduction of the scFv fragment was performed prior to the second thiol–maleimide reaction between the free maleimide groups of PEG4-DM1 (or PEG4-DM4) and the C-terminal cysteine of the protein. To this end, TCEP (tris(2-carboxyethyl)phosphine) was added in 0.5 mM concentration to 2 mg/mL of the scFv fragment in buffer A. The solution was incubated for 90 min at RT and with agitation, followed by the removal of the reducing agent using Zeba Spin Desalting Columns, 7 K MWCO (Thermo Fisher Scientific, Waltham, MA, USA). Immediately after that, PEG-DM1 (or PEG4-DM4) and the scFv fragment with the reduced C-terminal cysteine were mixed, so that the final molar ratio of PEG4-DM1 (or PEG4-DM4) to the scFv fragment constituted 1:2. This thiol–maleimide reaction was carried out for 16 h at 4 °C and with agitation. After the reaction, the products were purified from PEG4-DM1 (or PEG4-DM4) that did not react with the scFv fragment, and also partially purified from the unconjugated scFv molecules, using Zeba 7 K MWCO Columns and Amicon 30 kDa filters. The pegylated antibody fragment–drug conjugates, scFv-PEG4-DM1 or scFv-PEG4-DM4, were then sterilized through 0.22 μm membrane filters. The concentration of the scFv fragments in the conjugate solution was calculated at a wavelength of 280 nm using a BioDrop μLITE spectrophotometer (BioChrom, Cambridge, UK).

The production of pegylated scFv fragment conjugates without DM1 and DM4 drugs (scFv–PEG4) was performed as described previously [17]. A similar pegylation reaction was used to generate the fluorescent probe scFv-PEG4-FITC by conjugation with the FITC-SH reagent (Biopharma PEG Scientific Inc., Watertown, MA, USA). The scheme of the reaction is shown in Figure S1. Following a mild reduction of the C-terminal cysteine of the scFv fragment, reagents were added to the reaction mixture in buffer A according to the scFv: PEG4: FITC-SH molar ratio = 2:1:6. After incubation for 4 h at RT, filtration through Zeba Spin Desalting Columns and Amicon Ultra 30 kDa filters was performed to remove the free scFv and FITC-SH molecules that did not react with PEG4. The concentration of scFv fragments in the scFv-PEG4-FITC conjugate solution was calculated as described above.

### 2.2. SDS-PAGE

The pegylation efficiency was evaluated using polyacrylamide gel electrophoresis (SDS-PAGE), as described before [18]. For reducing electrophoresis, proteins in a standard SDS sample buffer containing 50 mM of dithiothreitol were heated to 95 °C for 5 min, and loaded onto the gels. The samples were resolved in 10% gels (NuPAGE Mini Protein Gels, Thermo Fisher Scientific, Waltham, MA, USA) and the gels were stained with Coomassie R250 and analyzed in Gel Doc EZ Imager and Image Lab software (Bio-Rad, Hercules, CA, USA).

### 2.3. Drug–scFv Fragment Ratio within the Pegylated Conjugates

The average drug–antibody ratio of the DM1 or DM4 drugs to the scFv fragments in the reaction product was calculated using ultraviolet–visible (UV-VIS) spectroscopy on a BioDrop µLITE spectrophotometer, as described by Chen [19]. The absorbance values at 253 nm wavelength, which was the absorption maximum for DM1 or DM4, and at 280 nm were used for calculating this ratio. PEG absorption is practically absent at these wavelengths and does not introduce errors into the calculation. The extinction coefficients employed in the analysis are presented in Table S1.

### 2.4. Direct ELISA

Nunc MaxiSorp high protein-binding capacity 96-well ELISA plates (Thermo Fisher Scientific) were coated with ganglioside GD2 (Sigma-Aldrich, Rockville, MD, USA) at a concentration of 0.1 μg in 100 μL of ethanol per well. Following air drying, the plate wells were blocked with 100 μL of 2% BSA in PBS-T per well for 2 h at RT. The parental scFv fragments or pegylated antibody fragment–drug conjugates (100 μL per well in PBS-T) were added in triplicate at different concentrations. Following incubation for 1.5 h at RT and washing with PBS-T, HRP-labeled anti-FLAG antibodies (1:6000) (Sigma-Aldrich) were added to the wells. After 40 min of incubation at RT and further washing, 1-Step Ultra TMB-ELISA Substrate Solution (Thermo Fisher Scientific) was added to the wells, and the color reaction optical density (OD) was measured at 450 nm using a Multiscan FC microplate reader (Thermo Fisher Scientific).

### 2.5. Cell Lines

The B16 and B78-D14 mouse melanoma cell lines were cultured in RPMI-1640 supplemented with 10% heat-inactivated fetal bovine serum, 2 mM of *L*-glutamine, 100 μg/mL of penicillin, and 100 U/mL of streptomycin (all—Thermo Fisher Scientific, Waltham, MA, USA).

The B16 cell line was obtained from ATCC. The GD2-positive B78-D14 mouse melanoma cell line generated by the transfection of the GD2-negative B16 line with genes coding for GD3 and GD2 synthases [20] was a kind gift from David Schrama (University Hospital Wuerzburg, Germany). The cell lines were maintained at low passage numbers and routinely checked for Mycoplasma using PCR.

### 2.6. Flow Cytometry

The staining of the B16 and B78-D14 cells with FAM-labeled GD2-specific antibodies ch14.18 (mAb-FITC) and scFv-PEG4-FITC was performed as described previously [21]. The mAb-FITC was obtained using the method described earlier [9], while the scFv-PEG4-FITC was generated in this work. In brief, the cells were detached from the culture plates, and were then incubated with mAb-FITC or scFv-PEG4-FITC (1 µg per sample) for 1 h in PBS supplemented with 1% FBS and 0.02% sodium azide. After incubation, the cells were washed twice in PBS. The relative fluorescence intensity (RFI) was calculated as the ratio of the specific fluorescence of the cells stained with mAb-FITC or scFv-PEG4-FITC to the autofluorescence of the control unstained cells.

For the staining of the B78-D14 cell line with unlabeled scFv fragments and pegylated conjugates that carried the octapeptide FLAG-tag, an additional incubation with FITC-labeled anti-FLAG antibodies (1:200) (Sigma-Aldrich) for 1 h was carried out, followed by an additional wash in PBS. The RFI was calculated as the ratio of the specific fluorescence of the cells stained with parental scFv fragments or the pegylated conjugates to the autofluorescence of the cells stained with FITC-labeled anti-FLAG antibodies.

All the procedures were performed at 4 °C. The samples were immediately analyzed using a BD FACSCalibur flow cytometer (Becton Dickinson, Franklin Lakes, NJ, USA). In each sample, at least 10,000 events were collected. For all the samples, the analysis was performed in triplicate. The data were analyzed using the FlowJo v10.9 and WinMDI 2.8 software.

### 2.7. MTT Assay

An analysis of the cytotoxic effects of the molecules was performed using a colorimetric MTT (3-[[4,5]-dimethylthiazol-2-yl]-2,5-diphenyltetrazolium bromide; purchased from Sigma-Aldrich) assay previously described by Denizot and Lang [22], with modifications described earlier [23]. Briefly, the B16 or B78-D14 cells were cultured in 96-well flat-bottom tissue culture plates (10^4^ cells/well, Greiner, Austria), with serial dilutions of the parental scFv fragments or the pegylated conjugates for 72 h under standard culture conditions. Following incubation, the MTT solution (final concentration 250 μg/mL) was added to each well for 4 h, after which, the precipitated formazan crystals were dissolved in DMSO. The reaction OD was assessed using a Multiscan FC microplate reader at a wavelength of 540 nm. The growth inhibition was calculated using the formula 100 − (OD _treated cells_ − OD _blank_)/(OD _control cells_ − OD _blank_) × 100%, where OD _blank_ represents the OD in the wells without cells. Dose–response curves were generated using SigmaPlot software (Systat Software Inc., Richmond, CA, USA). All the MTT experiments were reproduced at least three times.

### 2.8. Statistical Analysis

Graphs were created using SigmaPlot and MS Excel software. The data are represented as the mean ± SEM of at least three independent experiments, or as one representative experiment from three. The statistical analysis was performed by unpaired Student’s *t*-test. Significance levels of *p* < 0.05 were considered to be statistically reliable.

## 3. Results

### 3.1. Generation and Analysis of Pegylated FDCs

The tubulin polymerization inhibitors maytansinoids DM1 and DM4, alongside the auristatins MMAE and MMAF, are the most commonly used drugs for the generation of ADCs [24]. In addition, DM1 and DM4 are thiol-containing derivatives of maytansine, which allows for the use of maleimide chemistry to create theconjugates (Figure 1). Earlier, we successfully obtained multimeric fragments of GD2-specific antibodies by conjugating them to maleimide-activated PEG molecules with different molar mass and number of maleimide groups [17]. Here, in order to generate pegylated conjugates of scFv fragments of GD2-specific antibodies with the drugs DM1 or DM4, the 4-arm PEG-maleimide with a 10 kDa molecular weight (PEG4) was used, which enabled the simultaneous incorporation of the scFv fragment molecules and small-molecule inhibitors into a single chemical structure.

Due to the low solubility of DM1 and DM4 in aqueous solutions, the reaction was carried out in two stages. In the first stage, DM1 (or DM4) was mixed with PEG4 in 75% THF at a molar ratio of 12:1. This ratio allowed for most of the maleimide groups of PEG4 to be conjugated to DM1 (or DM4). A similar approach, but for a heterofunctional PEG molecule and the conjugation of DM1 to a full-length antibody, was used by Zhao et al. [25] and later by Hartimath et al. [26]. In the second thiol–maleimide reaction, the DM1-PEG4 or DM4-PEG4 conjugates purified from the unreacted components were mixed with the scFv fragments immediately after a mild reduction of their C-terminal cysteines. In contrast to the first reaction, the second reaction proceeded in an aqueous solution. The basic reaction scheme is shown in Figure 1.

**Figure 2:**
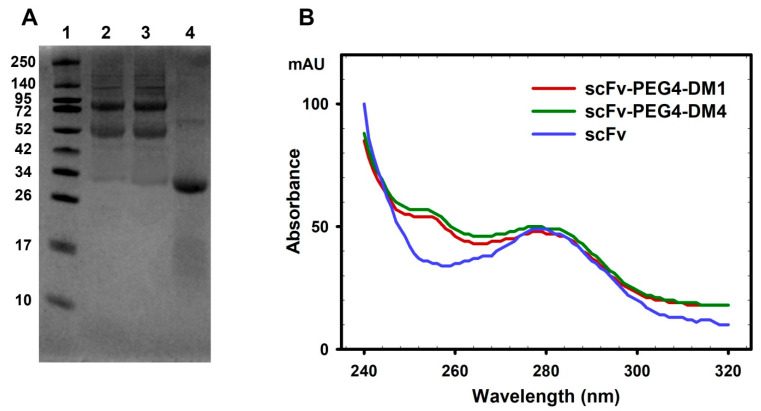
The analysis of the pegylated antibody fragment–drug conjugates. (**A**) Polyacrylamide gel electrophoresis of the pegylated conjugates and parental scFv fragment. 1, molecular weight protein markers; 2, scFv-PEG4-DM1; 3, scFv-PEG4-DM4; and 4, scFv. (**B**) Representative absorption spectra of the scFv-PEG4-DM1 and scFv-PEG4-DM4 fragment–drug conjugates, together with the unconjugated scFv fragment, all normalized at 280 nm.

The specific conditions of the second-stage reaction and the following purification of the products using filtration and size-exclusion chromatography allowed for the separation of the scFv-PEG4-DM1 and scFv-PEG4-DM4 conjugates from both the unconjugated small-molecule inhibitors DM1 or DM4, and the PEG4-DM1 or PEG4-DM4 conjugates that had a molar weight considerably smaller than the 30 kDa filtration cutoff of the filters. The same strategy enabled a partial purification from the unreacted scFv fragment of the GD2-specific antibody 14.18 with a 27 kDa molar weight. The protein products of the second-stage thiol–maleimide reaction were analyzed in 10% reducing polyacrylamide gel electrophoresis (SDS-PAGE) (Figure 2A). Pegylated scFv fragments constituted the predominant part of the protein bands, and most of them were pegylated scFv monomers and dimers with observed molecular weights of about 52 and 80 kDa, respectively (Figure 2A). It should be noted that pegylated proteins are characterized by an anomalous mobility in polyacrylamide gel compared to unmodified proteins of identical molecular weight, which is typically attributed to the bigger hydrodynamic radius of polyethylene glycol compared to that of proteins [27,28]. The positions of the bands of the pegylated scFv fragments are in full agreement with the corresponding bands in SDS-PAGE and the western blots obtained from our earlier work with scFv–PEG4 [17].

The average drug–antibody ratio of DM1 or DM4 to the monomeric scFv in the purified solution, as evaluated using UV-VIS spectroscopy, constituted 1.5 ± 0.2 and 1.6 ± 0.3 (in between *n* = 3 batches for each FDC, presented as mean ± SEM), respectively (Figure 2B, Table S1). While these ratios could not be applied directly to scFv-PEG4-DM1 and scFv-PEG4-DM4 due to the presence of a small amount of non-conjugated scFv in the final product, the multi-step purification warranted that all the DM1/DM4 was conjugated to the pegylated scFv molecules.

### 3.2. Antigen-Binding Properties of the Pegylated FDCs

**Figure 3:**
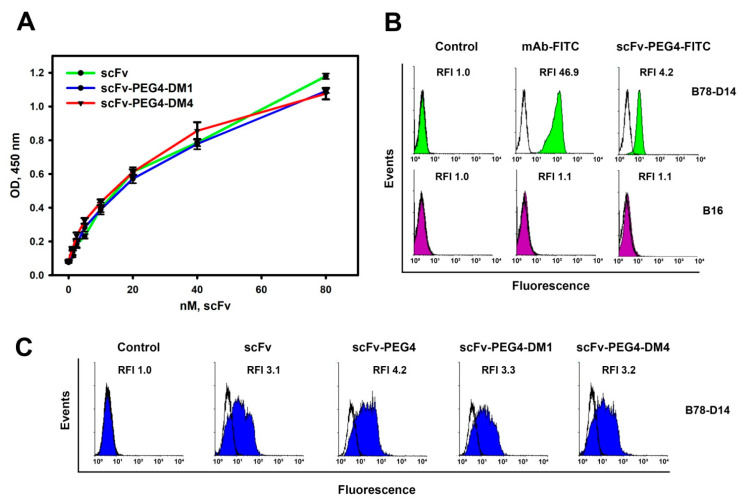
Antigen-binding properties of the pegylated antibody fragment–drug conjugates. (**A**) Evaluation of the binding of the pegylated conjugates and parental scFv fragments to ganglioside GD2 in direct ELISA. Data are presented as mean ± SEM. (**B**) Flow cytometry analysis. Staining of B78-D14 and B16 cell lines with mAb-FITC and scFv-PEG4-FITC. Control—fluorescence intensity of intact cells. MAb—GD2-specific antibodies 14.18. (**C**) Flow cytometry analysis. Incubation of B78-D14 cell line with unlabeled scFv fragments and conjugates, and staining with FITC-labeled anti-FLAG antibodies. Control—fluorescence intensity of cells stained with FITC-labeled anti-FLAG antibodies only.

The preservation of the antigen-binding properties of the resulting scFv-PEG4-DM1 and scFv-PEG4-DM4 conjugates was confirmed in direct ELISA. Ganglioside GD2 was adsorbed on the plate, and serial dilutions of the scFv fragments and obtained conjugates were added to the wells. The binding efficacy was determined using HRP-labeled anti-FLAG antibodies, specific to the FLAG-Tag incorporated into the structure of the parental scFv fragments, and by subsequent developing of the TMB colorimetric reaction (Figure 3A).

The dependence of the optical density (OD) on the concentration of pegylated conjugates or parental scFv fragments, represented in molar concentration of scFv, demonstrated that the conjugation to the pegylated DM1 or DM4 did not affect the GD2-binding properties of the antibody fragment (Figure 3A). This result was largely expected, since the pegylated multimeric scFv fragments which we obtained before using the PEG4 molecule demonstrated not only the preservation of the antigen-binding properties, but also the effect of avidity for the resulting conjugate, enhancing the binding to GD2 [17]. Moreover, the scFv fragments conjugated to the MMAE and MMAF drugs through the MC-Val-Cit-PAB linker also retained their binding to GD2 [9]. Such a short linker did not increase the solubility or stability of the conjugate in contrast to the PEG4 molecule used in present work [17].

To confirm the ability of the obtained conjugates to bind to GD2-positive cells, we generated the fluorescent probe scFv-PEG4-FITC, which was structurally similar to the scFv-PEG4-DM1 and scFv-PEG4-DM4. The reaction scheme of the generation of the scFv-PEG4-FITC is shown in Figure S1. The GD2-negative mouse melanoma cell line B16 and its derivative cell line B78-D14 overexpressing GD2 were used for flow cytometry staining. The FAM-labeled antibodies 14.18 (used as the control) and scFv-PEG4-FITC manifested strong binding to the GD2-positive B78-D14 and did not bind to the GD2-negative B16 cell line (Figure 3B). The lower fluorescence intensity of the GD2-positive cells stained with the scFv-PEG4-FITC compared to the cells stained with mAb-FITC can be explained by the larger number of fluorophore molecules in the fluorescent probe of the full-length antibody. As mentioned above, a FLAG-tag was included into the structure of the scFv fragment. In order to provide additional confirmation of the binding of the pegylated FDCs to GD2-positive cells, the staining of the B78-D14 cells with the original scFv fragment and its pegylated conjugates was performed, followed by secondary staining with FITC-labeled anti-FLAG antibodies (Figure 3C). As can be seen from the figure, the B78-D14 cells could be stained with all the scFv-containing molecules used in the work, including the pegylated FDCs, through detection with FITC-labeled anti-FLAG antibodies. Thus, it can be concluded that the obtained conjugates, scFv-PEG4-DM1 and scFv-PEG4-DM4, fully retained the ability of the parental scFv fragments to bind ganglioside GD2, and that conjugates with such a structure can specifically bind to GD2-positive cells.

### 3.3. Cytotoxic Effects of the Pegylated FDCs

**Figure 4:**
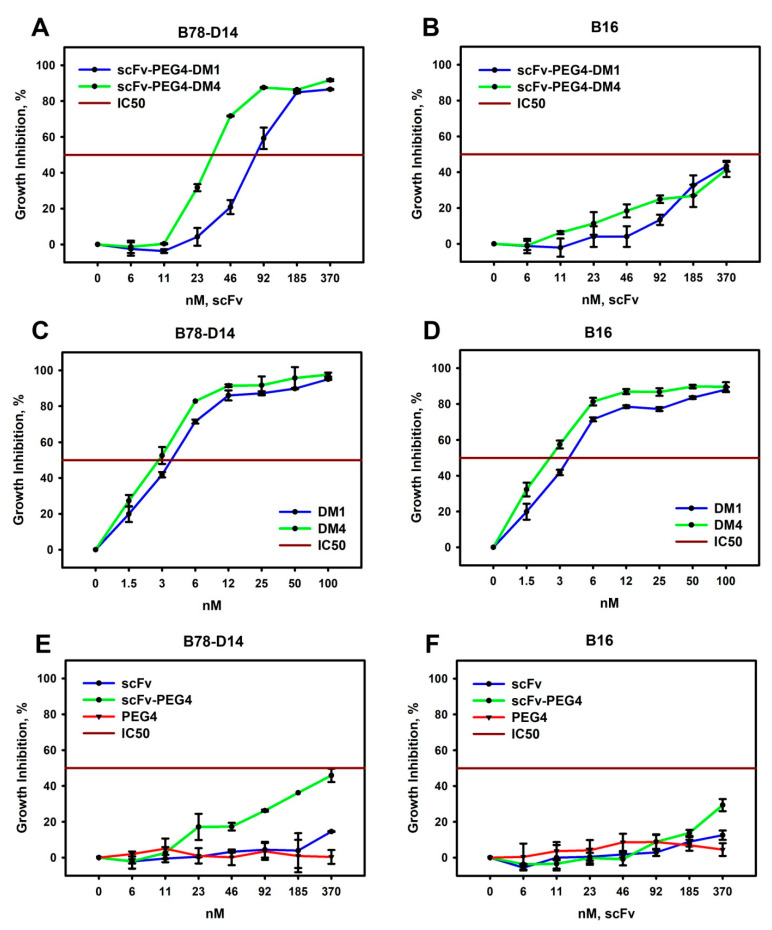
Cytotoxic activity of pegylated antibody fragment–drug conjugates in GD2-positive B78-D14 and GD2-negative B16 cell lines. Viability of mouse melanoma cell lines analyzed using MTT assay following 72 h incubation with scFv-PEG4-DM1 and scFv-PEG4-DM4 (**A**,**B**); DM1 and DM4 (**C**,**D**); and scFv, scFv-PEG4, and PEG4 (**E**,**F**).

The pair of the B78-D14 and B16 cell lines, differing mainly in GD2 expression [20], were used for a viability analysis after treatment with the obtained pegylated FDCs in the MTT assay. ScFv-PEG4-DM1 and scFv-PEG4-DM4 induced significant inhibitory effects in the B78-D14 cell line, in contrast to the GD2-negative B16 cell line (Figure 4A,B; *n* = 3, *p* < 0.05). Remarkably, scFv-PEG4-DM4 was more effective than scFv-PEG4-DM1 in inhibiting cell growth (Figure 4A). In the B78-D14 cell line, the IC50 of scFv-PEG4-DM4 was 34.1 ± 9 nM, whereas the IC50 of scFv-PEG4-DM1 constituted 80.2 ± 14 nM. Thus, the difference in the effectiveness of the obtained conjugates clearly depended on the drug—the cytotoxicity was approximately twice as high for the FDC loaded with DM4 as opposed to that with DM1 (Figure 4A; Table S2; Figure S2A). Both pegylated FDCs manifested considerably weaker effects in the B16 cell line that does not express GD2, inducing growth inhibition by 43.4 ± 2.1% and 41.4 ± 2.9% for scFv-PEG4-DM1 and scFv-PEG4-DM4, respectively, at the highest evaluated concentration (370 nM) (Figure 4B). This result indicates selective activity of the pegylated FDCs in GD2-positive cells.

At the same time, the free drugs did not demonstrate selective activity in GD2-positive and GD2-negative tumor cell lines; specifically, the IC50 of DM1 and DM4 in the B78-D14 cell line was roughly equal, 4.3 ± 0.3 and 3.1 ± 0.5 nM, respectively (Figure 4C,D, Table S2). It is noteworthy that the cytotoxicity of DM4 was slightly higher than the cytotoxicity of DM1 (Figure 4C,D). This data could explain the higher cytotoxicity of scFv-PEG4-DM4 compared to scFv-PEG4-DM1. It should also be noted that PEG4 had no effect on the viability of both cell lines used in this work (Figure 4E,F).

In our earlier work [17], pegylated multimeric scFv fragments of GD2-specific antibodies, in contrast to the parental scFvs, were capable of inducing direct cell death in GD2-positive cell lines. Therefore, here, we also evaluated the cytotoxic effects of the scFv fragments and pegylated multimeric fragments without DM1 or DM4 (Figure 4E,F). Unlike the parental scFv fragment, scFv–PEG4 was able to induce direct cell death in the GD2-positive B78-D14 cells. As the toxicity of scFv–PEG4 itself was much less prominent compared to the effect of the maytansine-bearing conjugates (significantly different in the B78-D14 cell line at IC50 concentrations of the FDCs; Figure 4A,E; *n* = 3, *p* < 0.05), the main cytotoxic activity of the scFv-PEG4-DM1 and scFv-PEG4-DM4 should be provided, respectively, by DM1 and DM4.

The cytotoxicity induced by the scFv-PEG4-DM1 and scFv-PEG4-DM4 conjugates, as well as by DM1, DM4, the parental scFv fragment, and the pegylated multimeric scFv fragment, in a different GD2-positive mouse lymphoma line EL-4 were very similar to the effects of these molecules in the GD2-positive B78-D14 cells (Figure S2, [17]).

## 4. Discussion

As a tumor marker, ganglioside GD2 is characterized by favorable characteristics, such as a wide distribution on different types of cancer, a high surface expression on tumor cells, and, unlike other gangliosides, a limited expression on healthy cells. This molecule is practically absent on healthy cells, except for minor expression on brain cells, peripheral nerves, and skin melanocytes. During tumor transformation, the expression of GD2 increases by orders of magnitude, reaching 10 million molecules per cell [1,29,30]. Hence, the prospects for developing targeted immunotherapy of GD2-positive tumors are obvious and promising. The vast number of both clinical trials and experimental studies regarding the various approaches of GD2-targeted therapy underscore the significant scientific interest in the topic. Currently, GD2-targeted therapy in the clinic is represented by two drugs, dinutuximab and naxitamab, both full-length GD2-specific antibodies. Both drugs are approved for the treatment of high-risk neuroblastoma. Several clinical trials are currently underway for selecting combinations with chemotherapy drugs, optimizing their regimens, and expanding their target indications [31,32].

A large number of studies on GD2-targeted therapy in formats other than full-length antibodies are being carried out, primarily justified by the insufficient effectiveness of “naked” full-length GD2-specific antibody therapy. The most promising areas include adoptive T- and NK-cell therapy, bispecific antibodies, immunocytokines, and vaccines, as well as the development of radiopharmaceuticals and antibody–drug conjugates (ADCs) [2,33].

At present, ADCs occupy a minor place within GD2-targeted therapy research, although the potential of this approach is significant, and it has shown considerable success in the treatment of other types of tumors, both in the clinic and preclinical trials. In the case of GD2-positive cancers, all of which are solid tumors, antibody FDCs are of particular interest. FDCs typically penetrate into solid malignant neoplasms better, accumulate in tumors in larger amounts in less time, and are able to eliminate tumors more efficiently, due to the smaller size of antibody fragments compared to full-length IgG molecules [3,7].

The aim of this work was to generate conjugates of scFv fragments of GD2-specific antibodies with DM1 and DM4 drugs using multivalent PEG molecules containing maleimide-activated groups, which would effectively serve as a platform for conjugating antigen-binding and effector molecules. We successfully obtained pegylated conjugates of scFv fragments of GD2-specific antibodies with DM1 or DM4 using a 4-arm PEG-maleimide with a 10 kDa molecular weight, which allowed for the incorporation of several molecules of scFv fragments and small-molecule inhibitors into a single chemical structure. The protein products of the second-stage thiol–maleimide reaction, analyzed using gel electrophoresis, showed that the pegylated scFv fragments constituted the predominant part of all the protein bands, and most of them were pegylated monomers and dimers. Based on the UV-VIS spectroscopy analysis, the ratios of the DM1 and DM4 drugs to the scFv fragments within the obtained conjugates were approx. 1.5 and 1.6, respectively. Taken together, a rough estimate could be made from combining the UV-VIS spectroscopy and gel electrophoresis data, that, in the resulting scFv-PEG4-DM1 and scFv-PEG4-DM4 conjugates, on average, 1.5 scFv molecules and 2.5 drug molecules were attached to one tetravalent PEG molecule.

The scFv-PEG4-DM1 and scFv-PEG4-DM4 fully retained the ability of the parental scFv fragments to bind ganglioside GD2 in direct ELISA and GD2-positive cells in flow cytometry analysis. These conjugates induced much stronger inhibitory effects in the GD2-positive B78-D14 cell line, in contrast to the GD2-negative B16 cell line. Interestingly, scFv-PEG4-DM4 was about twice more effective than scFv-PEG4-DM1 in decreasing the cell viability, probably due to the slightly higher cytotoxicity of free DM4 than that of DM1. Unlike the parental scFv fragments, the scFv–PEG4 conjugate was able to induce direct cell death in the GD2-positive B78-D14 cells. However, the cytotoxic activity was low compared to that of the FDCs in the selected concentration range; therefore, the main contribution to the overall cytotoxic activity of scFv-PEG4-DM1 and scFv-PEG4-DM4 was mediated by DM1 and DM4.

DM4 is considered by some to be more potent than DM1 in the context of delivery by ADCs due to its structural difference to DM1. An additional dimethyl group next to the terminal cysteine (see Figure 1) is present in DM4, which slightly increases the hydrophobicity of DM4 and facilitates its penetration into cells, thus increasing its cytotoxic effect, as well as bystander cell killing upon its antibody-mediated delivery to antigen-expressing cancer cells, compared to DM1 [34]. As of September 2023, DM1 and DM4 are components of two clinically approved ADCs, trastuzumab emtansine and mirvetuximab soravtansin, respectively, and while the difference in their cytotoxic activity is a topic of strong interest, their efficacy and side effects as payloads for antibody–drug conjugates largely depend on the antibody–drug linker type, targeted cancer, and antigen type [35].

We used pegylation as a common strategy to increase the in vivo half-life of therapeutic molecules. This strategy was justified in our work, since the molecular weights of both the scFv vector protein and the maytansinoids DM1 and DM4 were low and limited their therapeutic potential. The increased half-life of pegylated proteins is generally attributed to a much larger increase in the hydrodynamic radius (Rh) of the modified molecule compared to the addition of a protein with an equivalent molecular weight [27]. To date, at least 10 pegylated proteins have been approved for clinical use [15], including the anti-TNFa Fab fragment, certolizumab pegol, for the treatment of Crohn’s disease and rheumatoid arthritis. Due to its conjugation with the Y-shaped 40 kDa PEG, the half-life of certolizumab pegol in humans constitutes 2 weeks and is comparable to that of full-length antibodies [36]. Both the length of the PEG chain and the degree of its branching increase the circulation time of pegylated proteins. One study identified the8-arm 40 kDa PEG-maleimide as the best strategy for the intravitreal long-acting delivery of the anti-complement factor D Fab fragment, by comparing, among other parameters, the Rh of Fab–PEG conjugates that carried 1-, 2-, 4-, or 8-arm 40 kDa PEG-maleimides [37]. In our earlier study [17] regarding the multimerization of GD2-specific scFv fragments via pegylation, we observed a strong dependence of both the PEG length and the number of arms (branching) on the circulation time in vivo. Specifically, the blood-to-background ratio of the product of the scFv and 4-arm 10 kDa PEG-maleimide (abbreviated as scFv–PEG4 in this work) in mouse circulation constituted approx. 3.4 at 48 h post-injection, compared to approx. 1.3 for the scFv conjugated to monovalent 10 kDa PEG-maleimide. Importantly, the tumor uptake of scFv–PEG4 in the syngeneic EL-4 lymphoma mouse model at 24 h post-injection was higher than that of the naked scFv fragment and thecorresponding ch14.18 parent antibody [17].

Balancing the in vivo half-life of pegylated proteins carrying PEG chains of different lengths and numbers of arms with the degree of accumulation of the molecules in the tumor represents a different important topic of research, since an increase in the molecule size generally leads to a slower accumulation of the molecules in solid tumors. Specifically, Li et al. [11] demonstrated that conjugating the 5T4 antigen-specific diabody to a 20 kDa PEG with an Rh of 6 nm had superior tumor uptake compared to a conjugate carrying a 40 kDa PEG with an Rh of 12 nm, while both had comparable clearance kinetics. Ultimately, striking a balance between favorable pharmacokinetics, tumor distribution, and antitumor effects in vivo is of crucial importance for advancing FDCs into the clinic [38]. A more detailed study regarding the choice of PEG molecules could also be performed for the FDCs generated in this work. Such a study could include both the selection of the optimal length of the PEG chain and its number of arms. Employing PEG molecules with alternative functional groups, including heterofunctional variants, could increase the conjugation efficiency and product homogeneity, as well as simplify the purification of pegylated molecules.

The pegylated antibody fragment–drug conjugates developed using our approach may have certain advantages over standard ADCs or FDCs. Multivalent PEG molecules allow linking several of both the target and effector molecules that will likely retain their functional activity, through the formation of stable bonds. Pegylation leads to a better solubility of hydrophobic low-molecular-weight drug molecules and increases the EPR effect. Additionally, the proposed format of the antibody fragment–drug conjugate may exhibit reduced side effects compared to the two approved GD2-directed ADCs due to the absence of the Fc fragment in its structure and, hence, no complement-mediated cytotoxicity [39]. Thus, our original approach can contribute to the development of an important novel FDC format for the treatment of cancer.

## Supplementary Materials

The following supporting information can be downloaded at: https://www.mdpi.com/article/10.3390/cimb45100512/s1. Figure S1. Reaction scheme of the generation of the fluorescent probe scFv-PEG-FITC. Figure S2. Cytotoxic activity of pegylated antibody fragment-drug conjugates in GD2-positive mouse T-cell lymphoma cell line EL-4. Viability of EL-4 cell line analyzed by MTT assay following 72 hours incubation with scFv-PEG4-DM1 or scFv-PEG4-DM4 (A), DM1 or DM4 (B). GD2-positive mouse lymphoma EL-4 cell line was cultured in RPMI-1640. Cell line was kindly provided by Dr. A. Buzdin (Shemyakin-Ovchinnikov Institute of Bioorganic Chemistry, Russian Academy of Sciences). Table S1. Extinction coefficients employed in the analysis of the ratio of drugs to scFv fragments in the pegylated conjugates. Table S2. The cytotoxic activity of pegylated antibody fragment-drug conjugates and drugs in GD2-positive B78-D14 and EL-4 cell lines and in GD2-negative B16 cell line.
