# Supplementary material for: Targeting GD2-Positive Tumor Cells by Pegylated scFv Fragment–Drug Conjugates Carrying Maytansinoids DM1 and DM4
Source: Curr Issues Mol Biol. 2023 Oct 5;45(10):8112–25. doi: 10.3390/cimb45100512 (PMC10604934; doi:10.3390/cimb45100512)
Supplement: Supplementary file 1 [file cimb-45-00512-s001.zip › cimb-2606948-supplementary.pdf]

Targeting GD2-Positive Tumor Cells by Pegylated scFv Fragment–Drug Conjugates Carrying Maytansinoids DM1 and DM4

Supplementary Data

Table S1.
Extinction coefficients employed in the analysis of the ratio of drugs to scFv fragments in the pegylated conjugates.

**Molecule**	**Extinction coefficients (cm^{-1} M^{-1}) calculated at given wavelength**	
	**253 nm**	**280 nm**
scFv 14.18	26460	39960
DM1	26820	6560
DM4	21700	5140

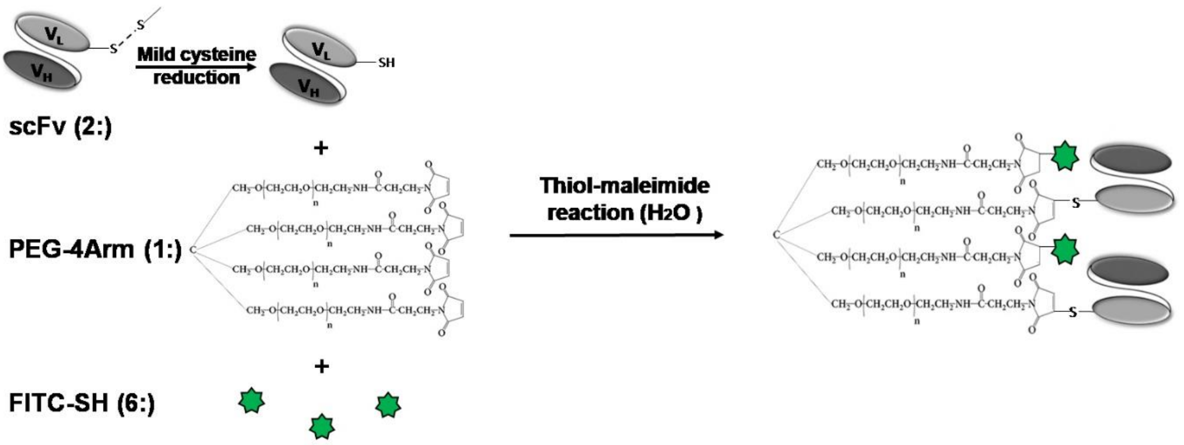

Figure S1. Reaction scheme of the generation of the fluorescent probe scFv-PEG-FITC

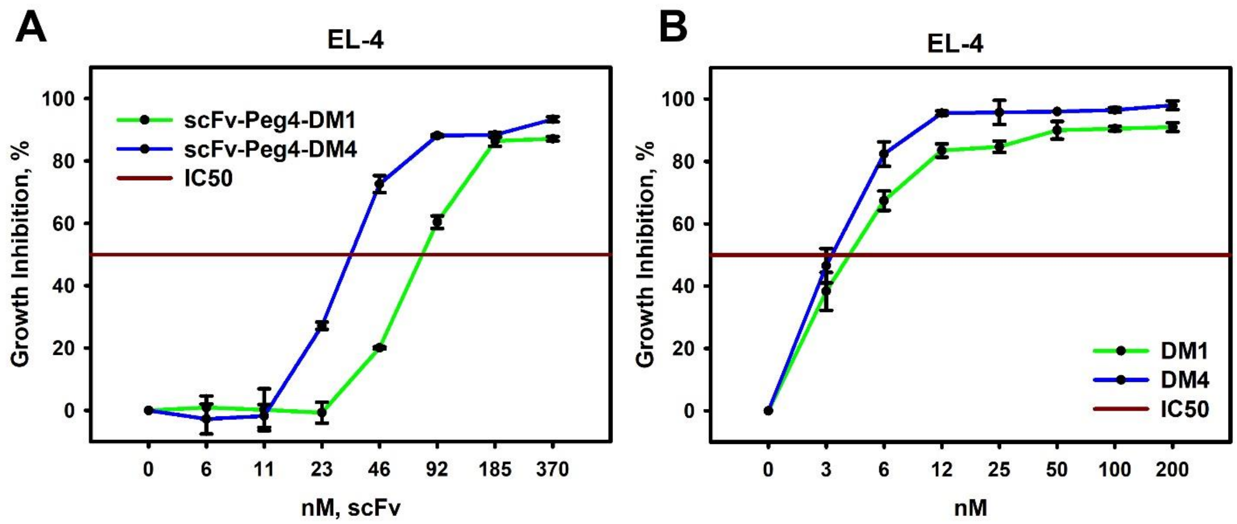

Figure S2. Cytotoxic activity of pegylated antibody fragment-drug conjugates in GD2-positive mouse T-cell lymphoma cell line EL-4. Viability of EL-4 cell line analyzed by MTT assay following 72 hours incubation with scFv-PEG4-DM1 or scFv-PEG4-DM4 **(A)**, DM1 or DM4 **(B)**. GD2-positive mouse lymphoma EL-4 cell line was cultured in RPMI-1640. Cell line was kindly provided by Dr. A. Buzdin (Shemyakin-Ovchinnikov Institute of Bioorganic Chemistry, Russian Academy of Sciences).

Table S2. The cytotoxic activity of pegylated antibody fragment-drug conjugates and drugs in GD2-positive B78-D14 and EL-4 cell lines and in GD2-negative B16 cell line.

	IC50 nM			
	scFv-PEG4-DM1	scFv-PEG4-DM4	DM1	DM4
B78	80.2±14	34.1±9	4.3±0.3	3.1±0.5
B16	-	-	4.2±0.5	2.7±0.4
EL4	79.9±18	34.5±12	4.9±0.9	3.2±0.3
